# Single‐Cell Atlas Reveals Neutrophil Heterogeneity in Hypervirulent *Klebsiella pneumoniae* Infection‐Induced Sepsis

**DOI:** 10.1111/imm.70193

**Published:** 2026-08-27

**Authors:** Xiang Fang, Weizhao Li, Xinyu Zhang, Sohana Akter Mina, Qianling Jiang, Gaochen Zhu, Sheng Chen, Guan Yang

**Affiliations:** ^1^ Department of Infectious Diseases and Public Health, Jockey Club College of Veterinary Medicine and Life Sciences City University of Hong Kong Hong Kong China; ^2^ State Key Lab of Chemical Biology and Drug Discovery and the Department of Food Science and Nutrition The Hong Kong Polytechnic University Hong Kong China

**Keywords:** bacterial infection, heterogeneity, hypervirulent *Klebsiella pneumoniae*, neutrophil, sepsis

## Abstract

Hypervirulent *Klebsiella pneumoniae* (hv*Kp*) infection often induces sepsis characterised by remarkable phenotypic and functional diversity among neutrophils. However, the roles of distinct lung neutrophil compartments involved in hv*Kp* infection remain poorly understood. Here, utilising single‐cell RNA sequencing, we identified four neutrophil subsets in the lungs of hv*Kp*‐infected mice: N1, N2, N3a, and N3b, which were organised along a maturation trajectory. Functionally, early‐stage subsets exhibited proinflammatory and phagocytic activities with high levels of activation, whereas terminal subsets displayed enhanced pathogen sensing and immunoregulatory properties. Notably, the N3b population showed pronounced immunosuppressive features and was predicted to inhibit lymphocyte responses through Lgals9 and Ceacam1 ligands, potentially associated with hv*Kp*‐induced lymphopenia. Projection onto NeuMap further validated the functional states of identified subsets, linking N1 to proliferative programmes, N2 to granules and hypoxia‐associated metabolic activity, N3a to antigen‐presentation signatures and N3b to immunosuppressive hubs. Morphologically, N3a/b were predicted to be enriched for segmented neutrophils, consistent with a more mature phenotype compared with N1 and N2. Notably, segmented neutrophils were markedly expanded in infected lungs, suggesting their association with hv*Kp* pathogenesis. Together, our findings delineate the lung neutrophil landscape in hv*Kp*‐induced sepsis, providing insights for understanding hv*Kp* pathogenesis and developing targeted therapeutic interventions at a neutrophil subset level.

## Graphical abstract

The roles of distinct lung neutrophil compartments involved in hv*Kp* infection remain poorly understood. Here, utilising single‐cell RNA sequencing, we identified four neutrophil subsets in the lungs of hv*Kp*‐infected mice: N1, N2, N3a, and N3b, which were organised along a maturation trajectory. Functionally, early‐stage subsets exhibited proinflammatory and phagocytic activities with a high level of activation, whereas terminal subsets displayed enhanced chemotaxis‐related and immunoregulatory properties.

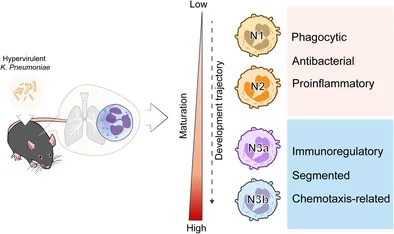

## 1 Introduction

*Klebsiella pneumoniae* (*Kp*) is a gram‐negative commensal and opportunistic bacterium of the *Enterobacteriaceae* family. It can cause many detrimental infectious diseases in humans, especially in immunocompromised or critically ill individuals [1]. A major concern is the emergence of hypervirulent *K. pneumoniae* (hv*Kp*), which exhibits markedly greater virulence than classical strains and has increasingly been associated with severe infections over the past three decades. Despite significant advances in our understanding of hv*Kp*, mortality remains high due to complications such as immune dysregulation, including cytokine storm and lymphopenia [2, 3]. Therefore, elucidating host immune responses to hv*Kp* infection is essential for identifying potential therapeutic targets.

Neutrophils play a critical role in eliminating invading bacteria and serve as the main defence at the early stages of infection. Accumulating evidence suggests that neutrophils are not a homogeneous population; rather, they exhibit substantial transcriptional and functional heterogeneity, characterised by diverse maturation states and specialised subsets with distinct transcriptomic profiles [4], as well as immunosuppressive capabilities that inhibit T‐cell‐mediated host defence [5]. However, the heterogeneity of neutrophils in hv*Kp*‐infected lungs, and their characterisations including precise expression programmes, developmental dynamics, functions, and maturation states, remain poorly understood.

In this study, we utilised single‐cell RNA sequencing (scRNA‐seq) to dissect the transcriptional features of distinct neutrophil populations in hv*Kp*‐infected lung through unbiased approaches. Our study defines the neutrophil landscape in hv*Kp*‐induced sepsis, providing insights for understanding subset‐specific contributions to hv*Kp* pathogenesis.

## 2 Materials and Methods

### 2.1 Bacterial Strain and Culture Condition

The hv*Kp* (17ZR101) strain was described in our previous publication [3]. The bacteria were cultured in Luria‐Bertani medium with shaking at 220 rpm at 37°C and Luria‐Bertani agar medium at 37°C overnight as previously described [6].

### 2.2 Animal Experiment

All animal experiments were approved by the Animal Ethics Committee of the City University of Hong Kong (AN‐STA‐00000194) and followed the guidelines of the Institutional Laboratory Animal Research Unit. Six‐ to eight‐week‐old female mice were used in the study. For the infection model, mice were intravenously injected with ~5 × 10^3^ CFU of 17ZR101 strain. At 12 hpi, lung, spleen, bone marrow and blood samples were collected for flow cytometry analysis, Haematoxylin and Eosin (H&E) staining and Immunofluorescence.

### 2.3 Flow Cytometry

Lung tissues were excised and incubated in HBSS with 1× HEPES and 0.04 mg/mL Collagenase type I at 37°C. After 1‐h digestion, the tissues were filtered through a 70‐μm strainer. Blood samples were collected in 0.5 M EDTA via orbital enucleation. Red blood cells were lysed with ACK lysing buffer (Thermo Fisher, A1049201). Dead cells were labelled using ghost dye violet 510 (Tonbo Biosciences, 13‐0870‐T100) or Propidium Iodide (Sigma‐Aldrich, 25535‐16‐4). Fluorescently labelled monoclonal antibodies against CD45 (BioLegend, 103112, 103114), CD11b (BioLegend, 101226), Ly6G (BioLegend, 127607), Ly6C (eBioscience, 17‐5932‐82), CD177 (BD Pharmingen, 566599) and PD‐L1 (BioLegend, 124313) were used for cell surface staining. For chemotaxis assay, cells were labelled with CXCL2 antibody after fixation and permeabilisation with Intracellular Fixation & Permeabilisation Buffer Set (eBioscience, 88‐8824‐00) for 1 h. For neutrophil activation testing, cells were stained with CD63 following the instructions. All of the labelled samples were tested by a BD FACSCelesta flow cytometer (BD Bioscience). Data were further analysed by FlowJo software (Version 10.8.1). Flow cytometry gating strategies are shown in Figure S1.

### 2.4 Histological Experiment and Immunofluorescence Staining

The left lower lung tissues were collected after infiltrating with 1 × PBS from uninfected and infected mice and fixed in 4% paraformaldehyde overnight, followed by dehydration and paraffin embedding. Paraffin‐embedded tissues were sectioned at 5‐μm, mounted on glass slides and stained with H&E staining (Solarbio, G1120). For immunofluorescence staining of neutrophils, lung sections were deparaffinised and rehydrated, followed by boiling in a Tris EDTA (Beyotime, P0084) for antigen retrieval. Next, tissue slides were incubated with blocking buffer (3% BSA and 0.1% TritonX‐100 in PBS) at room temperature for 1 h and stained with anti‐Ly6G antibody conjugated with PerCy5.5 in blocking buffer (1:100) in the dark for 1 h, followed by three washes. DAPI was then stained at 1:1000 for 1 h. All lung histological images were acquired using a Nikon Laser Scanning Confocal Microscope and then processed with ImageJ software.

For neutrophil morphology analysis, single‐cell suspensions were prepared from lung, spleen, bone marrow and peripheral blood as described for flow cytometry. Cells were centrifuged onto glass slides using cytospin at 700 g for 5 min. Cytospin preparations of cell suspension were fixed and stained using a Bio‐Diff Kit (BioGnost) according to the manufacturer's instructions. To characterise neutrophil nuclear morphology in tissues, cells were categorised into four distinct phenotypes based on established criteria: preneutrophils (PreNeu), identified by banded nuclei and a high nuclear‐to‐cytoplasmic ratio; immature neutrophils (ImmNeu), exhibiting banded nuclei with a low nuclear‐to‐cytoplasmic ratio; segmented neutrophils (SegNeu), defined by the presence of two to five nuclear lobes and hypersegmented neutrophils (HyperSegNeu) with more than five nuclear segments [7].

### 2.5 Neutrophil Functional Assay

Single‐cell suspensions were prepared from infected mouse lungs via enzymatic digestion, filtration and red blood cell lysis as described in Section 2.3. To assess neutrophil extracellular trap (NET) formation, cells were incubated with SYTOX Blue nucleic acid stain (Thermo Fisher Scientific, S11348) for 30 min at 37°C according to the manufacturer's instructions. For the evaluation of intracellular reactive oxygen species (ROS) levels, cells were loaded with a DCFH‐DA probe (Solarbio, CA1410) for 20 min at 37°C at the recommended working concentration. To determine phagocytic capacity, cells were co‐cultured with latex beads (Sigma, L0280) for 3 h at 37°C following the manufacturer's protocol. Subsequently, cells were stained with fluorochrome‐conjugated antibodies against specific cell‐surface markers prior to flow cytometric analysis.

### 2.6 Single‐Cell Data Preprocessing and Cell Type Determination

The single‐cell data was obtained from our previous publication [3] (GSE220594). Single‐cell suspensions of lung tissues were prepared from individual animals, with *n* = 1 mouse for the uninfected group and *n* = 1 mouse for the hv*Kp*‐infected group. All downstream analyses of this dataset were performed at the individual‐cell level to characterise transcriptional differences among cells. Samples from different animals were processed independently without pooling. Analysis of scRNA‐seq data was performed using Seurat (version 5.1.0) and SCP (version 0.5.6). The filter criteria included number of genes, mitochondrial RNA percentage and number of UMIs. A standard data integration process was performed by Seurat. After integration, data scaling, PCA, dimensionality reduction (using UMAPs) and clustering were applied for cluster identification and data visualisation. Cell markers for each neutrophil subset were identified using the FindAllMarkers function in Seurat. Differentially expressed genes with Benjamini‐Hochberg adjusted *p* < 0.05 and an expression frequency of > 50% within the target cluster were retained. The top 20 marker genes for each cluster were then selected according to the average log2 fold change (Table S1). To identify clusters, we searched the top five markers ordering by log‐fold change in CellMarker 2.0 (http://www.bio‐bigdata.center/index.html) and used the marker of established lung cell types to comprehensively classify the cells. Genes expressed with top log2 fold‐change, *p* < 0.05 and pct.1 > 0.3 were considered significantly differentially expressed. Differentially expressed genes (DEGs) were displayed using SCP 0.5.6.

### 2.7 Identification of Immunosuppressive Cells

To representatively evaluate the immunosuppressive features of neutrophil subsets, we scored the different subgroups using the conserved MDSC signature published previously [8] after reclustering neutrophils with new UMAP dimensions. All score analysis was conducted using Seurat's ‘AddModuleScore’ function. Multigroup comparisons were conducted using the Kruskal–Wallis Test in the default parameters of FeatureStatPlot (SCP 0.5.6).

### 2.8 Assessment of Neutrophil Functions

Neutrophil functional scores were calculated using the ‘AddModuleScore’ function in Seurat. Gene sets used for other scoring were obtained from relevant Gene Ontology (GO) biological pathways. Phagocytosis‐related genes were from GO:0006911. ROS genes were defined according to GO:1903409. Neutrophil activation score was evaluated based on genes related to GO:0042119. Metabolic glycolysis of subsets was scored with the expressions of #R‐MMU‐70171 pathways in Reactiome Pathway Database. Chemotaxis was assessed using genes annotated in GO:0030593. The detailed gene lists used for each functional score are provided in Table S2.

### 2.9 Developmental Trajectory Inference

Pseudo‐time was generated with Monocle 2 [9] to infer the potential lineage differentiation trajectory. The newCellDataSet function (lowerDetectionLimit = 0.1; expressionFamily = negbinomial.size) was used to build the object based on the genes of N1–N3b in hv*Kp* groups. The root group was chosen by maturation score and CytoTRACE method (Figure S2) [10].

### 2.10 Projection of External scRNA‐Seq Data

The original scRNA data downloaded from datasets were implemented using R (v.4.0.3) and the package Seurat (v.4.0.5). The Seurat objectives were analysed with the standard Seurat pipeline, and the filter criteria and markers for the definition of cell populations were followed by methods from corresponding publications [4, 11, 12]. Additionally, we projected our neutrophils onto Wang and Xie's data and annotated them with our custom labels using Seurat's FindTransferAnchors and TransferData, while the projection of NeuMap was conducted with MapQuery and TransferData to transfer our labels. Subset distributions were presented as areas containing the core 85% of cells, defined by the 15th percentile of the KMASS algorithm, which calculates the density of cells in specific areas with *n* = 100 grid points in each direction.

### 2.11 Cell Communication Analysis

Cell–cell communication analysis was performed using the CellChat R package. Briefly, Seurat‐normalised expression data and cell labels were integrated as the input. Ligand‐receptor interactions encompassing secreted, extracellular matrix (ECM) and cell–cell contact pathways were inferred across infected and uninfected groups using CellChatDB via the triMean approximation. To identify infection‐induced alterations, differential communication probabilities were evaluated between groups, and an ultra‐fast Wilcoxon rank‐sum test via the presto package was employed to determine significantly upregulated or downregulated signalling pairs with adjusted *p* < 0.05 among the targeted immune cell populations.

### 2.12 Statistical Analysis

The statistical analysis of data from this study was performed by Graphpad Prism 10.0. Normality of the data distribution was assessed using the Shapiro–Wilk test. Statistical comparisons of Ly6G^+^ populations in CD45^+^ cells between uninfected and infected groups were conducted using unpaired *t* test, while paired *t* test was applied to compare the CD63 MFI in CD177^hi^ versus CD177^low/−^ neutrophils and CXCL2 MFI in PD‐L1^+^ versus PD‐L1^−^ neutrophils. For single‐cell‐level data, statistical comparisons of functional scores across multiple neutrophil subsets were performed using the nonparametric Kruskal–Wallis test. To evaluate changes in neutrophil morphology across different anatomical sites following infection, the proportions of different neutrophil morphologies in the bone marrow, peripheral blood, spleen and lung were analysed using two‐way ANOVA followed by Sidak's multiple comparisons test.

## 3 Results

### 3.1 ScRNA‐Seq Reveals Distinct Neutrophil Clusters in hv*Kp* ‐Infected Lung

Reanalysis of our previously published scRNA‐seq data [3], complemented by flow cytometry and histological analysis, has revealed that hv*Kp* infection induces a pronounced myeloid expansion dominated by neutrophil accumulation (Figure 1a–c). scRNA‐seq revealed a total of 3701 neutrophils, including 645 neutrophils in the uninfected group and 3056 in the infected group (Figure 1f). To investigate the heterogeneity of neutrophils in hv*Kp* infection, we subclustered neutrophils into four transcriptionally distinct subsets (N1, N2, N3a, and N3b) based on their gene expressions (Figure 1d). The distribution of these subsets was further validated in a septic lung scRNA‐seq dataset [12] (Figure 1e). The relative proportions of the four neutrophil subsets differed between the sham and hv*Kp* datasets (Figure 1f), with N1 enriched in metabolic and respiratory pathways, N2 in synaptic vesicle endocytosis and N3a/b in innate immune activation (Figure 1g). Among the top‐ranked marker genes for each subset, the N1 population exhibited a potent capacity for peroxidase‐negative killing, characterised by secondary granule genes such as *Ltf* and *Camp* [13]. The N2 subset was distinguished by granule‐associated genes, including *Mmp8*, a pivotal granule protein in neutrophil host defence [14]. N3a showed marked expression of inflammation regulatory genes such as *Asprv1*, an aspartic protease associated with neutrophil‐mediated inflammation and immunosuppressive neutrophil accumulation [8, 15], whereas N3b was defined by the chemotaxis‐related genes, including *Ccl3*, crucial for neutrophil mobilisation (Figure 1h) [16].

**FIGURE 1:**
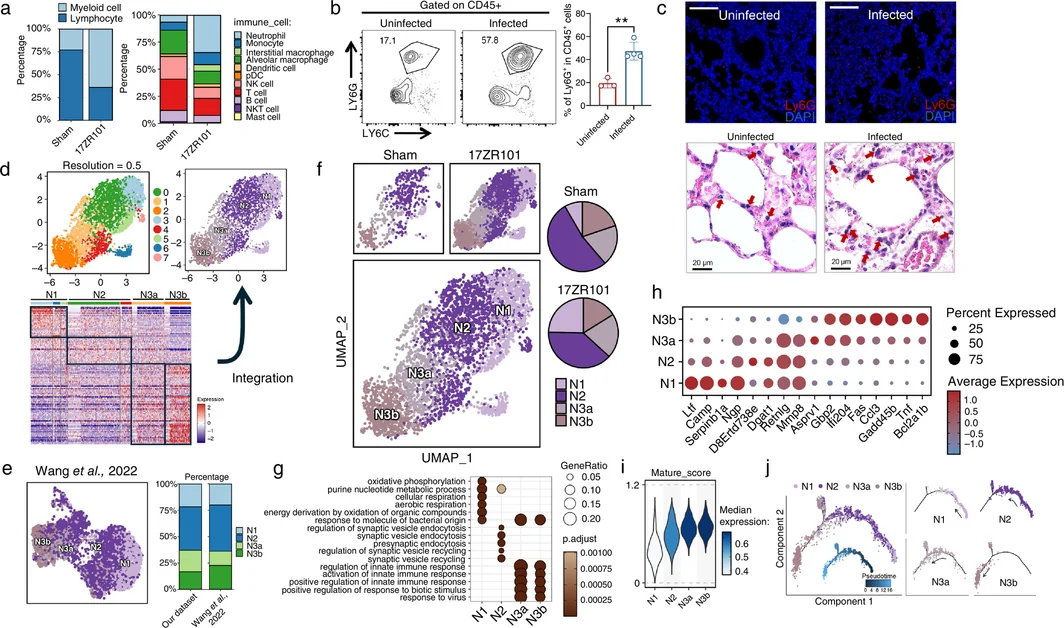
ScRNA‐seq analysis reveals distinct neutrophil subsets in the lung. (a) Proportions of different immune cells in hv*Kp*‐infected and uninfected group of scRNA data (GSE220594). (b) Flow cytometry analysis of frequencies of Ly6G^+^ cells in uninfected (*n* = 3) and hv*Kp*‐infected (*n* = 4) lungs. Data are mean ± SEM. Student's two‐tailed *t* test. **p < 0.01. (c) Histological images of lung samples from mice injected with 5000 CFU hv*Kp* or PBS and sacrificed at 12 hpi. Upper: Representative immunofluorescence images of lung samples stained with Ly6G and DAPI (bar = 100 μm). Bottom: Representative H&E images of lung samples with highlighted neutrophils (arrowheads) (bar = 20 μm). (d) Definition of neutrophil subsets based on transcriptional profiles. Top left: Clusters of neutrophils based on resolution of 0.5; Top right: Final annotation of neutrophil subsets after integration according to expression modules; Bottom: Heatmap depicting normalised expression of genes in each cluster. (e) Comparison of neutrophil subsets in our data with lung neutrophils identified by Wang et al. (GSE207651). (f) UMAP of neutrophils from lungs, coloured by inferred cluster identity (bottom), and distribution of neutrophil subsets in uninfected or infected group. (g) GO enrichment analysis of four neutrophil subsets. (h) Dot plot depicting the scaled expression of selected signature genes for each cluster. The colour shows the average expression level. Dot size represents the percentage of cells expressing the corresponding gene. (i) Score value of gene sets for neutrophil maturation. (j) Monocle trajectories of neutrophils (left) and respective trajectories of each subset (right).

To capture the whole spectrum of maturation status of these subsets, we measured the maturation score used by a previous study [4]. N3a and N3b populations were the most mature neutrophils, while N1 was the least mature, with N2 displaying an intermediate level (Figure 1i). Transcriptional trajectory analysis showed that the data are consistent with a putative maturation trajectory, starting from N1 and N2 neutrophils to terminal N3a and N3b subsets (Figure 1j).

### 3.2 Functional Diversity of Neutrophil Clusters in hv*Kp* ‐Infected Lung

Transcriptional differences of neutrophil populations suggest distinct functional specialisations. Functional pathway scoring revealed that N1 neutrophils exhibit high levels of phagocytosis, ROS production, activation and glycolytic activity (Figure 2a–d), highlighting their metabolically related antibacterial functions [17]. N2 neutrophils also show a strong capacity for phagocytosis but reduced ROS production, activation and glycolysis (Figure 2a–d), indicating a rewired metabolic state and metabolic reprogramming. Furthermore, the migratory potential of N3a and N3b was stratified by chemotactic scores, with N3a exhibiting an intermediate level and N3b displaying the highest score (Figure 2e).

**FIGURE 2:**
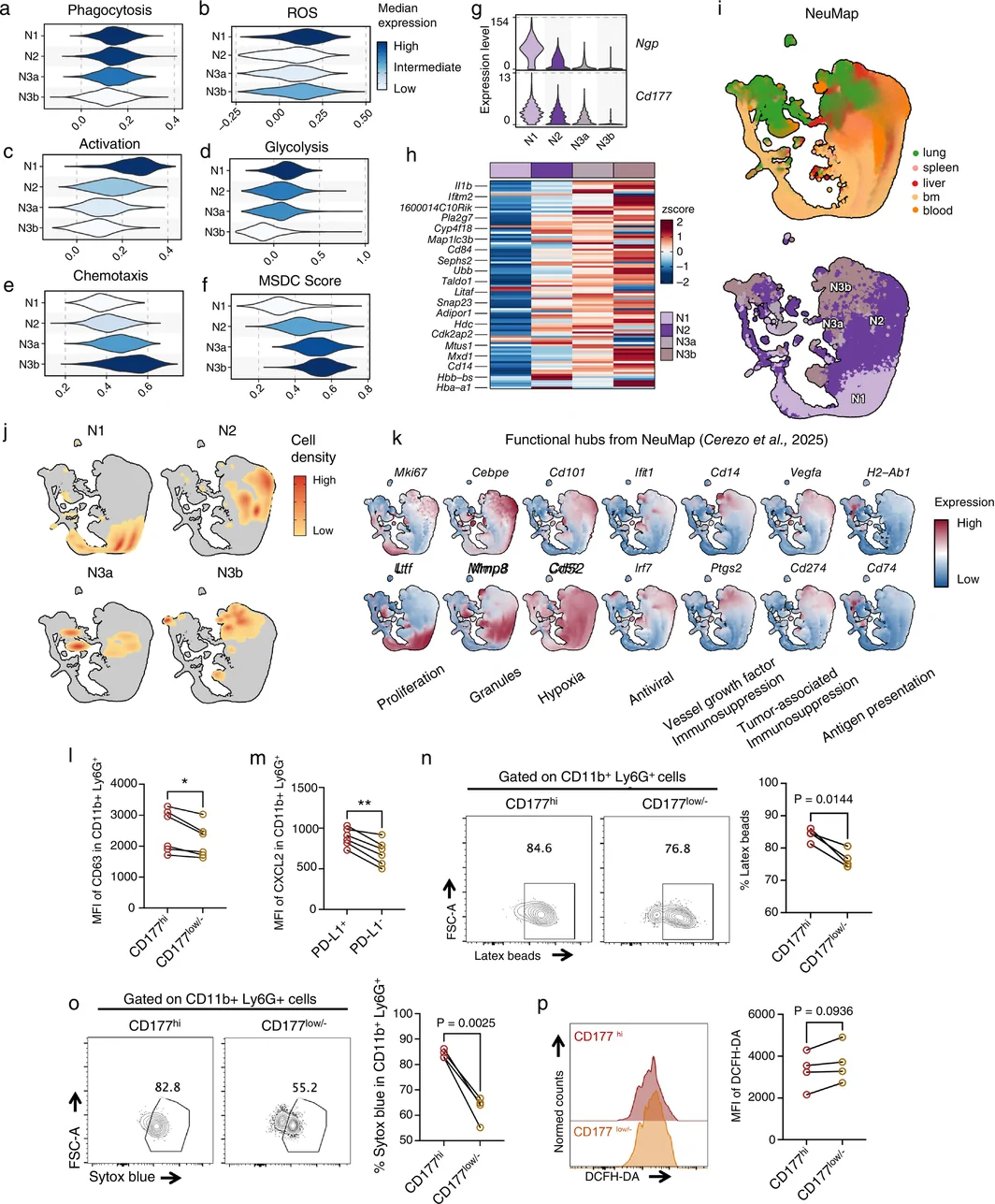
Functional characterisation of neutrophil subpopulations. (a–f) Violin plot of phagocytosis score (GO:0006911), ROS score (GO:1903409), neutrophil activation score (GO:0042119), metabolic glycolysis score (Reactome Pathway Database #R‐MMU‐70171), chemotaxis score (GO:0030593), and MDSC score for each cluster. (g) Expressions of inflammatory neutrophil markers. (h) Heatmap of MDSC‐related genes across neutrophil subsets. (i) Projection of neutrophils from our subsets to NeuMap (GSE266680). Left panel: UMAP of NeuMap. Right panel: Label transfer of our N1, N2, N3a, and N3b subsets to NeuMap. (j) Density plot showing distribution of neutrophils from our dataset in NeuMap. Clusters in figures are shown as the area with the accumulation of 85% of cells for each selected cluster. Cells were calculated using K‐MASS with confidence predictions over 0.7. (k) UMAP plots depicting marker distributions of functional hubs from NeuMap. (l and m) Mean fluorescence intensity (MFI) of CD63 and CXCL2 measured by flow cytometry, compared between CD177^hi^ and CD177^low/−^ counterparts, or PD‐L1^+^and PD‐L1^−^ neutrophils (*n* = 6). (n–p) Functional characterisation of CD177^hi^ and CD177^low/−^ neutrophils by flow cytometry, including phagocytosis, NET formation, and ROS production assays. Data are presented as mean ± SEM (*n* = 4). Paired *t* test was applied for comparisons. *p < 0.05, **p < 0.01.

Given that hv*Kp* infection is characterised by cytokine storm and lymphopenia, which were closely correlated with the proinflammatory neutrophils [3] and immunosuppressive neutrophils [5], we focused on characterising these phenotypes within the subsets. To evaluate their potential immunosuppressive properties, we applied an unbiased myeloid‐derived suppressive cell (MDSC) signature scoring method [8]. Our results showed that N3a/b exhibited the highest MDSC scores, suggesting potential immunosuppression‐associated features, whereas N2 exhibited intermediate levels, potentially representing transitional states. Notably, N3b expressed highest levels of immunosuppressive mediators, highlighting its strengthened suppressive potential (Figure S3). In contrast, N1 displayed the lowest score and higher levels of proinflammatory markers, reflecting a more classical neutrophil phenotype (Figure 2f–h). Alongside trajectory analysis, these results may imply a developmental transition from a proinflammatory to an immunosuppressive phenotype.

To gain a comprehensive understanding of different subsets, we reanalysed and projected our analysis onto the single‐cell neutrophil reference atlas (Figure 2i,j), NeuMap [11], which includes healthy homeostasis, acute bacterial/viral infections, sepsis, autoimmune diseases and various tumour models, with multiple tissues such as bone marrow, peripheral blood and inflamed peripheral organs. When referenced against functional markers, N1 aligned with proliferative hubs, consistent with a less mature state (Figure 2k); N2 displayed granules and hypoxia‐related signatures, supporting its metabolic reprogramming; N3a was enriched for antigen‐presentation programmes, highlighting their capacity for recognition of pathogens and N3b corresponded to immunosuppressive hubs, corroborating with our MDSC scoring. In addition, the functions of these clusters are stable and not resolution‐dependent, as refined clusters are still located in the same NeuMap hubs (Figure S4). Overall, this mapping supports our classification of neutrophil subsets.

Activated neutrophils are known to exhibit metabolically related antibacterial functions and degranulation. Consistently, proinflammatory N1 neutrophils showed the highest activation score, indicating potent antimicrobial capacity (Figure 2g). *CD177*, identified among the top differentially expressed genes of N1 subset encoding a surface marker, was selected as a representative marker for flow‐cytometric validation of the N1‐enriched population (Figure S5, Table S1). Results further confirmed that CD177^hi^ neutrophils expressed higher levels of activation marker CD63 compared to CD177^low^ and CD177^−^ counterparts (Figure 2l). Additionally, functional assays revealed that CD177^hi^ neutrophils exhibited significantly increased NET formation and enhanced phagocytic capacity, whereas ROS production remained comparable between groups, demonstrating that their robust antibacterial potential is primarily mediated through NETosis and phagocytosis. Neutrophil chemotaxis is highly associated with the CXCL family. PD‐L1^+^ neutrophils, representing an N3b‐enriched population identified based on *Cd274* expression (Figure S5, Table S1), significantly expressed CXCL2, an important member of the CXCL family responsible for neutrophil self‐recruitment [18] (Figure 2m), thereby supporting their increased chemotaxis‐associated features. This finding further suggests their potential role in enhancing immunosuppression by recruiting other suppressive neutrophils [19].

### 3.3 Cell–Cell Communication Highlights the Interaction Patterns of Immunosuppressive Neutrophils With T and B Cells

Neutrophil crosstalk with immune and tissue cells is essential in hv*Kp* infection, as it integrates various immune responses such as inflammatory amplification and immune suppression. Through cell chat analysis of scRNA‐seq data, we found that hv*Kp* infection increased both the number and strength of interactions among the immune cells (Figure 3a). Notably, the outgoing interactions of neutrophils with other immune cells were enhanced, highlighting their regulatory capacity in immune responses (Figure 3b). Given the observed lymphopenia in hv*Kp* infection [5], we investigated the presence of such suppressive interactions. Ligand‐receptor analysis suggested that neutrophil‐associated immunosuppressive signalling may involve inhibitory ligands LGALS9 and CEACAM1 [20, 21]. Rather than specific ligand‐receptor pairs, these signals primarily reflect interactions with Ighm B‐ and Cd8a T‐cell populations (Figure 3c). These signalling mechanisms were predictably mediated by the N3b subset, which exhibited the highest expression levels of the investigated suppressive ligands (Figure 3d).

**FIGURE 3:**
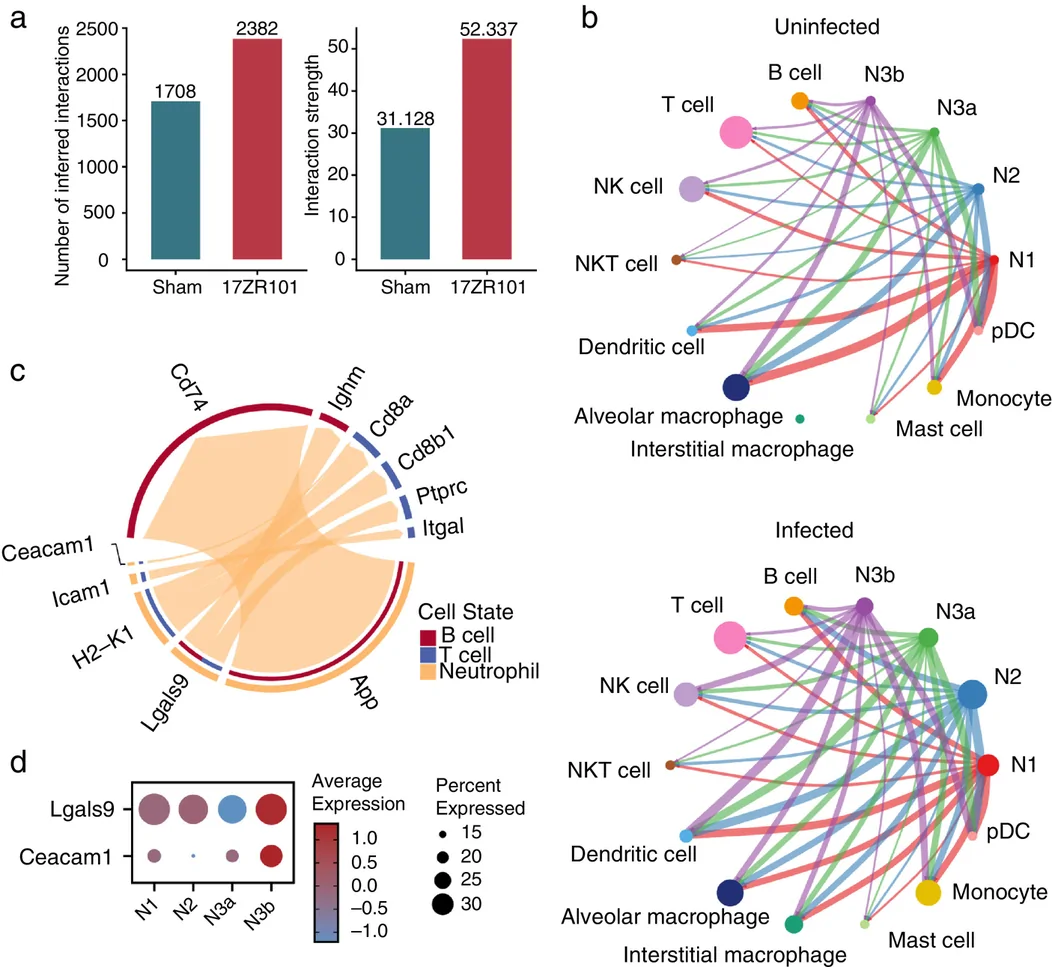
Cell communication highlights the interaction patterns of immunosuppressive neutrophils with T and B cells. (a) Bar plots showing total number and strength of cell–cell communications across all immune cell types. (b) The network of outgoing interactions from neutrophil subsets to other immune cells in uninfected (top) and hv*Kp*‐infected (bottom) conditions. Cell clusters show as nodes, with line thickness indicating changes in communication strength. (c) The chord plots of ligand‐receptor pairs from neutrophils to T and B cells. (d) Dot plot depicting expressions of suppressive ligands identified from (c) for each neutrophil cluster. Dot size represents the percentage of cells expressing the corresponding genes.

### 3.4 Correlation Analysis With Known Subpopulations Reveals the Morphological Features of Neutrophil Subsets

Neutrophil maturation is characterised by distinct morphological stages that reflect varying granular types and functions. In our study, we identified four nuclear phenotypes in the lungs: preneutrophil (PreNeu), characterised by banded nuclear and a high nuclear‐to‐cytoplasmic ratio; immature neutrophils (ImmNeu), which also exhibit a banded phenotype but with a low nuclear‐to‐cytoplasmic ratio; segmented neutrophils (SegNeu), exhibiting two to five nuclear lobes and hypersegmented neutrophils (HyperSegNeu), having more than five nuclear segments [7] (Figure 4a). To predict and align our scRNA neutrophil subsets with corresponding morphologies, we applied a cell label transfer method and inferred the populations based on transcriptomic similarities with previously published clusters that had identified morphological characteristics [4], where the G3 subset was identified as immature banded neutrophils mainly from bone marrow, whereas G4 and G5 represented mature segmented neutrophils existing in peripheral blood and spleen. According to this, our results indicated that N1 population corresponded to G3 and G4, while N2 comprised G4 and G5 lineages (Figure 4b,c). N3a/b were primarily associated with G5 populations. These results indicate that both N1 and N2 are inferred to comprise a mixture of banded and segmented cells encompassing PreNeu, ImmNeu, SegNeu, and HyperSegNeu, whereas N3a/b are predicted to predominantly consist of SegNeu and HyperSegNeu.

**FIGURE 4:**
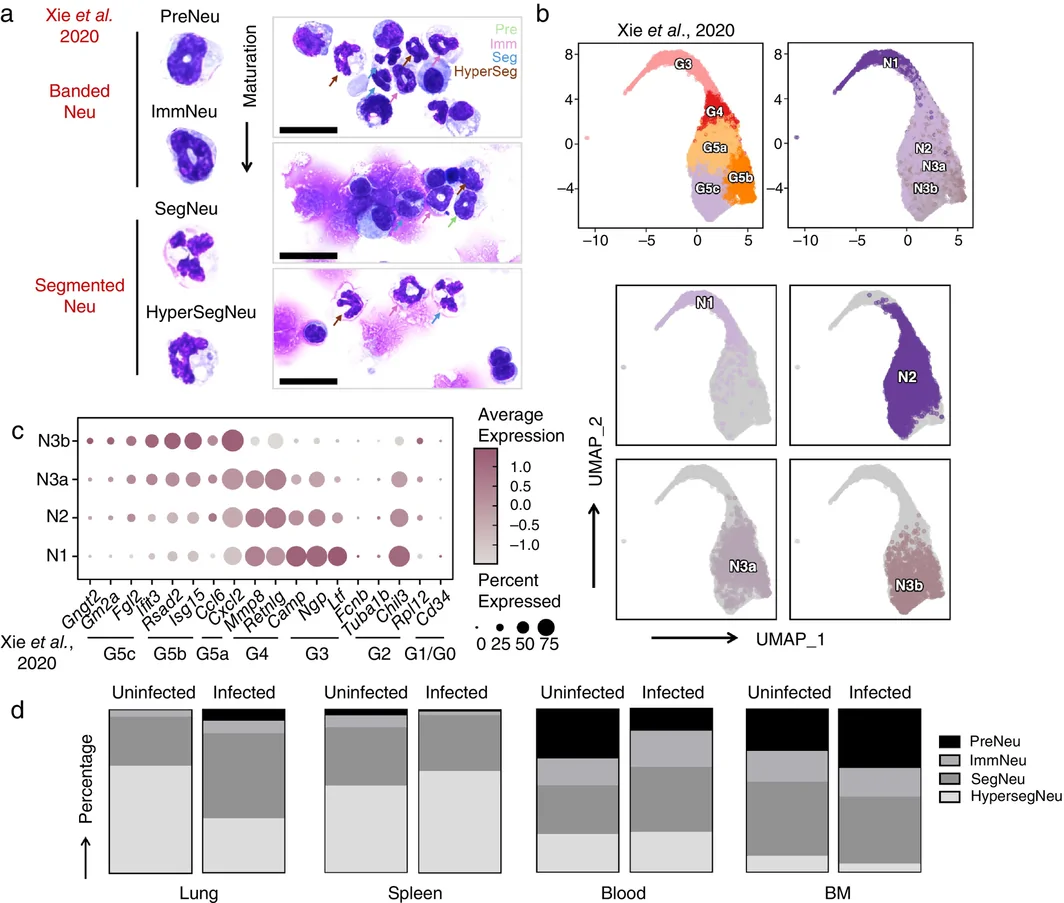
Correlation analysis with previously identified neutrophil subsets. (a) Diff‐quik staining of different neutrophils in the lung (bar = 20 um). PreNeu, ImmNeu correspond to the morphology of banded neutrophils described by Xie et al., while SegNeu and HyperSegNeu are segmented neutrophils. (b) Comparison of neutrophil populations in our data with subsets identified previously in Xie's data (GSE 137540). Cluster labels are transferred from our data to Xie's. Left: UMAP of distribution of original subsets and transferred subsets. Right: Distribution of our four subsets in Xie's neutrophil UMAP. (c) Dot plot illustrating the average expression levels of G3, G4, G5a/b/c marker genes in our N1, N2, N3a, and N3b subsets. Dot size represents the percentage of cells which express the genes. (d) Percentages of four morphological subsets in infected or uninfected lungs (*n* = 3/group), spleens (*n* = 4/group) and bone marrow (*n* = 4/group) samples. For each biological replicate, 100–200 neutrophils are manually calculated from multiple nonoverlapping fields of view. Morphological classification was performed independently and in a blinded manner by two investigators.

### 3.5 Morphological Heterogeneity of Neutrophil Subsets Across Different Tissues

Having mapped the transcriptional neutrophil states to nuclear morphology, we next investigated their tissue‐specific morphological heterogeneity during hv*Kp* infection. This heterogeneity is directly relevant to disease progression, given the established links between neutrophil maturation, effector function and infection‐driven immunopathology. Using cell smear, we assessed the morphological heterogeneity of neutrophils from infected and uninfected tissues. The lungs exhibited a marked shift toward terminal neutrophil maturation, characterised by a significant increase in the proportion of SegNeu along with a decline in HyperSegNeu (Figure 4d and Figure S6c,d), suggesting a significant emergency recruitment of freshly matured neutrophils to the site of infection. Meanwhile, the systemic circulation and haematopoietic reservoir showed distinct mobilisation patterns. In the blood, we observed a dramatic depletion of the PreNeu pool accompanied by a slight compensatory rise in SegNeu, suggesting rapid consumption and maturation of the circulating neutrophil reserve during acute infection (Figures 4d and S6b,c). Interestingly, the bone marrow responded by significantly increasing its PreNeu population, reflecting an emergency granulopoiesis to meet the high demand for cells in the infected peripheral tissues (Figure 4d).

## 4 Discussion

Understanding the cellular and molecular mechanisms underlying immune dysregulation induced by hv*Kp* is essential for enhancing current therapeutic strategies, particularly those aimed at modulating host immune responses. Myeloid cells, especially neutrophils, are highly heterogeneous, comprising both proinflammatory and immunosuppressive subsets that are linked to disease progression. While our previous study has characterised some markers and signalling pathways of pro‐inflammatory myeloid cells in hv*Kp*‐infected lungs [3], the full spectrum of neutrophil heterogeneity remains poorly understood. Specifically, beyond these pro‐inflammatory subsets, the expression profiles of other diverse clusters, cell interactions, developmental trajectories and maturation states of neutrophils during hv*Kp* infection‐induced sepsis remain unexplored. Here, to unravel the impact of different neutrophil subsets on hv*Kp* pathogenesis, we generated a single‐cell transcriptomic atlas of distinct neutrophil populations, along with their divergent maturation status and morphologies during hv*Kp* infection‐induced sepsis. This analysis aimed to dissect the unique transcriptomic and morphological features that differentiate various neutrophil counterparts.

In our transcriptional characterisation, the transcriptome of neutrophil subpopulation is organised in a directed structure, possibly reflecting the short lifespan of neutrophils. However, although this trajectory was defined based on maturation‐related features, activation‐associated changes may also contribute to the observed transcriptional continuum, as indicated by the gradual decline in activation scores along the trajectory. Other factors, such as tissue localisation or recruitment source, may further contribute to this heterogeneity [22]. Dissecting the relative contribution of these factors will require future spatial profiling and lineage‐tracing studies.

Although hv*Kp* infection‐induced sepsis is characterised by cytokine storm [3] and lymphopenia [5], how distinct neutrophil subpopulations contribute to these pathological features remains poorly understood. Our data reveal functionally specialised neutrophil subsets that are differentially linked to proinflammatory and immunosuppression during hv*Kp* infection. Through marker and functional analysis, we identified N1 and N3b subsets with distinct roles in infection: The N1 population expressed higher levels of secondary granule genes, and displayed enhanced activation, NET formation and phagocytosis with comparable ROS production, indicating its enhanced degranulation and antibacterial functions. However, this hyper‐activated state might lead to uncontrolled generation of neutrophil NETs and proinflammatory responses, ultimately resulting in tissue damage [23, 24]. Previous studies have shown that hyperactivated neutrophils can produce proinflammatory cytokines that amplify inflammatory responses [25, 26]. Therefore, the expansion of the N1 subset during hv*Kp* infection may contribute significantly to the cytokine storm associated with sepsis. However, cytokine production was not directly assessed in this study, and whether specific cytokines produced by N1 contribute to the cytokine storm warrants further investigation. Conversely, the N3b subset exhibits the highest level of chemotaxis‐related programmes and is representatively characterised by the expression of *Cd274* (encoding PD‐L1). Extensive evidence highlights the immunosuppressive functions of PD‐L1^+^ neutrophils on lymphocytes [27, 28]. The chemotactic activity of N3b may promote recruitment of lymphocytes and maintain spatial proximity, facilitating cell interactions essential for their potential inhibitory functions.

In our study, hv*Kp* infection disrupted the balance between the pro‐inflammatory N1 population and the immunosuppressive N3b subset. Under physiological conditions, immunosuppressive neutrophils are essential for restraining excessive inflammation and preventing uncontrolled immune responses [29]. However, during hv*Kp* infection, this regulatory balance may become insufficient to counteract the expansion and activation of N1 neutrophils, resulting in excessive inflammatory responses and pulmonary cytokine production [3, 30]. Meanwhile, the accumulation of N3b neutrophils may impair lymphocyte responses and contribute to immune suppression [31]. Ultimately, this imbalance between innate inflammatory activation and adaptive immune dysfunction may delay bacterial clearance and exacerbate severe sepsis progression.

Neutrophils are mediators and are responsible for immune dysregulation in various diseases. To better understand the cellular interactions contributing to hv*Kp*‐induced lymphopenia, we analysed neutrophil crosstalk with other immune cells. Our ligand‐receptor analysis predicted potential interactions between the N3b subset and lymphocytes involving Ceacam1 and Lgal9. Previous studies have reported that Ceacam1 can suppress lymphocyte activation through ITIM‐dependent signalling and cooperation with TIM‐3 [32], while Lgals9 can engage TIM‐3 on T cells and contribute to T‐cell depletion [33]. Together, these findings suggest that N3b neutrophils may exhibit immunosuppression‐associated features through Ceacam1 and Lgal9 signalling.

In our morphological analysis, we correlated the neutrophil subsets identified from scRNA‐seq analysis with various morphological types. Neutrophils with more nuclear lobes undergo chromatin remodelling and cytoskeletal reorganisation, reflecting their flexibility to migrate through tissue structures and respond rapidly to stimuli [34]. The N3a and N3b subsets, exhibiting more nuclear lobulation, may benefit from enhanced migration capabilities and access to lymphocyte‐rich niches, thereby promoting immunosuppressive interactions.

Our study highlights the neutrophil heterogeneity in hv*Kp* infections. However, several limitations warrant consideration. First, our study is limited by investigating the neutrophil heterogeneity in a single inflammatory condition. Perturbations associated with autoimmune diseases, virus infections and chronic diseases remain uncharted. Second, the scRNA‐seq analysis was based on a limited number of biological samples, and thus sample‐specific effects may not be excluded. Third, while we identified morphological differences across neutrophil subsets, the exact mechanisms underlying this divergence remain unclear. Finally, our study establishes a comprehensive landscape of neutrophils in hv*Kp* infection‐induced sepsis, providing insights for understanding hv*Kp* pathogenesis and developing targeted therapeutic interventions at the subset level.

## Author Contributions

**Xiang Fang:** methodology, formal analysis, investigation, writing – original draft, visualisation. **Weizhao Li:** methodology, writing – review and editing. **Xinyu Zhang:** methodology, data curation. **Sohana Akter Mina:** methodology. **Qianling Jiang:** writing – review and editing. **Gaochen Zhu:** writing – review and editing. **Sheng Chen:** conceptualisation, writing – review and editing, supervision, project administration, funding acquisition. **Guan Yang:** conceptualisation, writing – original draft, writing – review and editing, supervision, project administration, funding acquisition.

## Funding

This work was supported by the Innovation and Technology Fund (ITS/111/24) and the Theme‐Based Research Scheme (T11‐104/22‐R).

## Conflicts of Interest

The authors declare no conflicts of interest.

## Supporting information


**Figure S1:** Gating strategies for neutrophils, CD177^hi^ and PD‐L1^+^ subtypes.
**Figure S2:** CytoTRACE analysis reveals N1 as developmental root. Boxplot depicting differentiation potency of N1, N2, N3a, and N3b.
**Figure S3:** Expressions of inhibitory mediators in subsets. Dot plot depicting expressions of *Nos2* and *Tgfb1* for each neutrophil cluster. Dot size represents the percentage of cells expressing the corresponding genes.
**Figure S4:** Projection of fine‐grained neutrophil clusters onto NeuMap. (a) UMAP distributions of refined neutrophil clusters at resolution 0.5 (upper) and resolution 0.8 (middle) and the merged neutrophil subsets N1–N3b (bottom). (b) NeuMap projections comparing the clusters at resolution 0.5 and 0.8, with the merged neutrophil subsets N1–N3b. Cluster labels mark the centres of the corresponding cell groups. (c and d) Density plots showing the distributions of neutrophil clusters identified at resolutions 0.5 and 0.8, respectively, in NeuMap. Contours denote the areas encompassing 85% of cells.
**Figure S5:** Distribution of Cd177^+^ cells and Cd274^+^ cells. (a and b) UMAP plot showing the expression distribution of Cd177 and Cd274 in neutrophils. (c and d) Percentages of Cd177^+^ cells and Cd274^+^ cells in N1, N2, N3a, and N3b.
**Figure S6:** Comparison of neutrophil morphological proportions in different tissues. (a–d) comparisons of neutrophil morphologies (PreNeu, ImmNeu, SegNeu, and HypersegNeu) across distinct anatomical compartments under infected or uninfected conditions, including the bone marrow, peripheral blood, lung, and spleen. Data are presented as mean ± SD (*n* = 3–4 mice per group). Statistical significance was determined separately for each morphology using two‐way ANOVA with infection status and tissue type as factors, followed by Sidak's multiple comparisons test to compare infected and uninfected groups within each tissue. *p < 0.05, **p < 0.01, ****p < 0.0001.

**Table S1:** Top 20 gene markers of neutrophil subsets.
**Table S2:** Neutrophil functional score genes.

## Data Availability

The custom R analytical codes are accessible on GitHub (https://github.com/FangX636/ScRNA‐seq‐analysis.git). Processed Seurat object and associated metadata for re‐analysis of neutrophils have been deposited in our previously published dataset (GSE220594). The newly generated experimental data, including flow cytometry, morphological analyses and image‐quantification data, will be made available from the corresponding author upon reasonable request.
