# Supplementary material for: Single‐Cell Atlas Reveals Neutrophil Heterogeneity in Hypervirulent Klebsiella pneumoniae Infection‐Induced Sepsis
Source: Immunology. 2026 Aug 27;179(4):624–34. doi: 10.1111/imm.70193 (PMC13725439; doi:10.1111/imm.70193)
Supplement: Supplementary file 1 [file IMM-179-624-s001.pdf]

Supplementary Figures

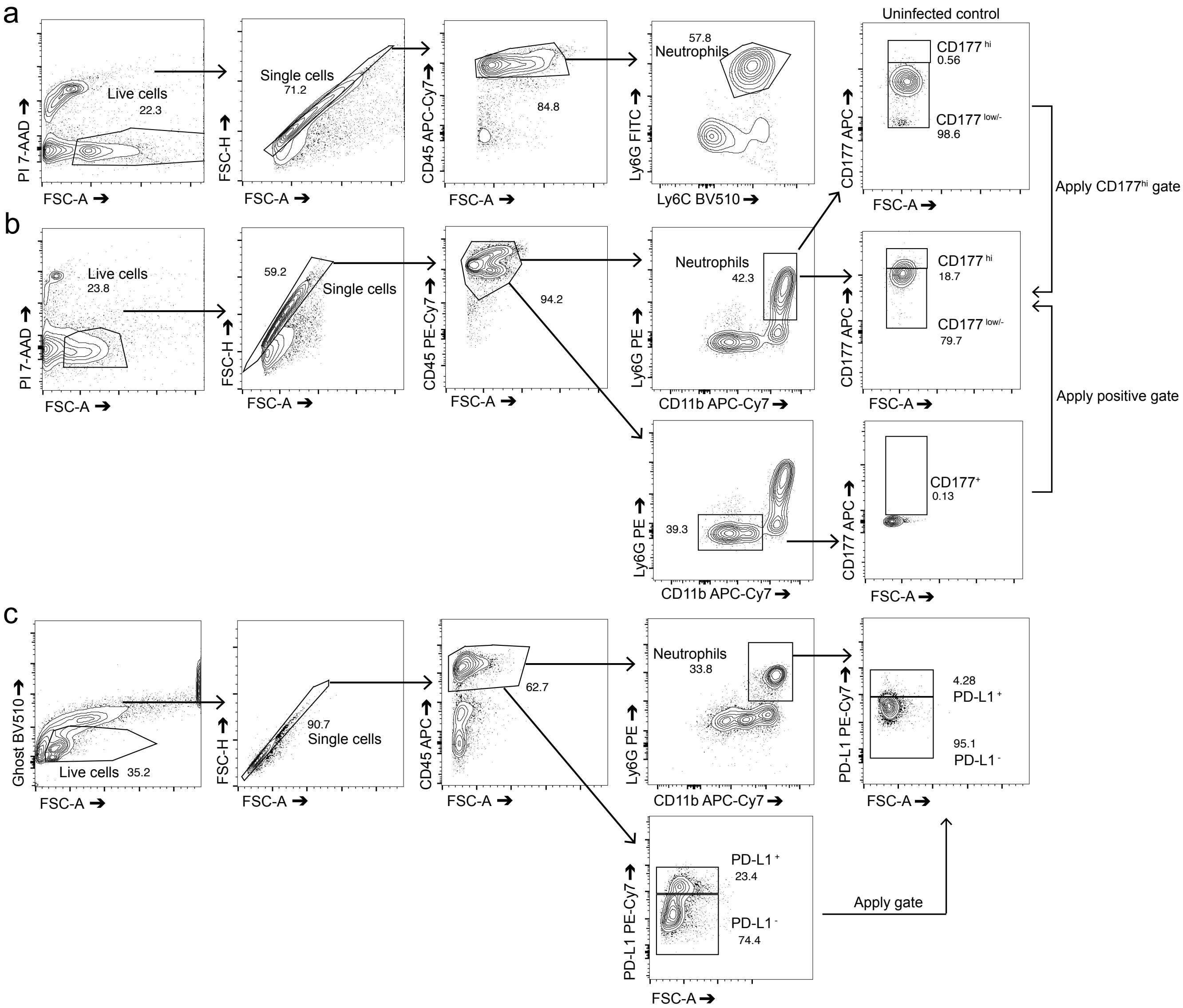

Fig. S1. Gating strategies for neutrophils, CD177^{hi} and PD-L1^{+} subtypes.

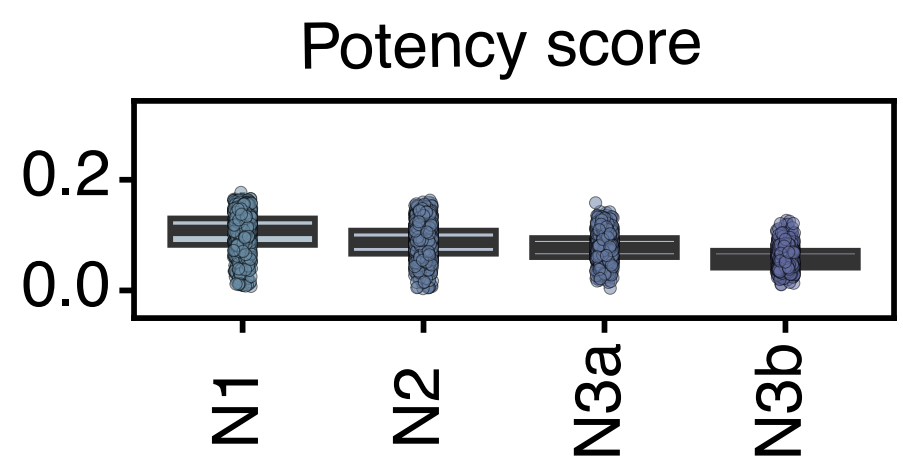

Fig S2. CytoTRACE analysis reveals N1 as developmental root. Boxplot depicting differentiation potency of N1, N2, N3a, and N3b.

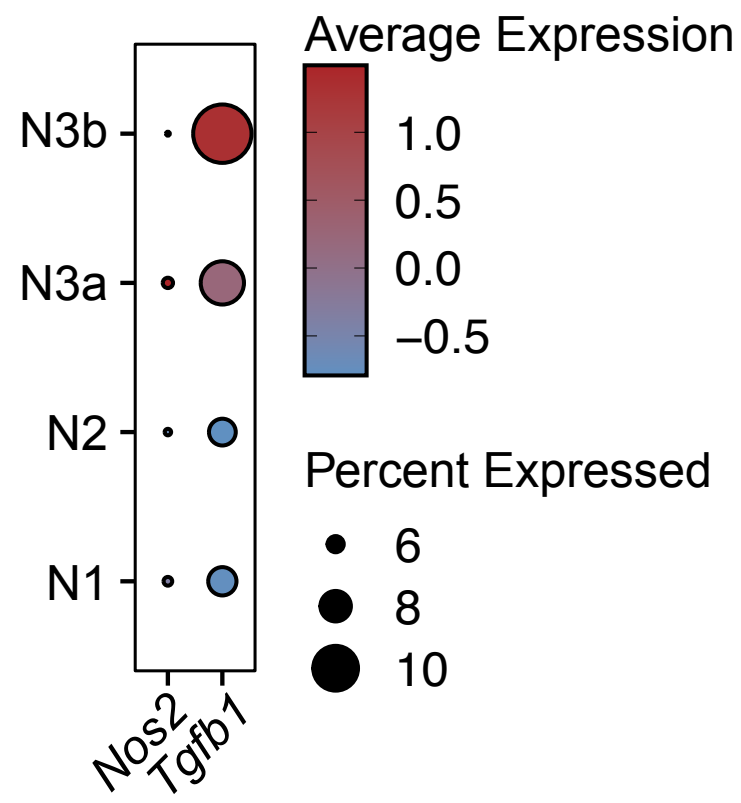

Fig. S3. Expressions of inhibitory mediators in subsets. Dot plot depicting expressions of *Nos2* and *Tgfb1* for each neutrophil cluster. Dot size represents the percentage of cells expressing the corresponding genes.

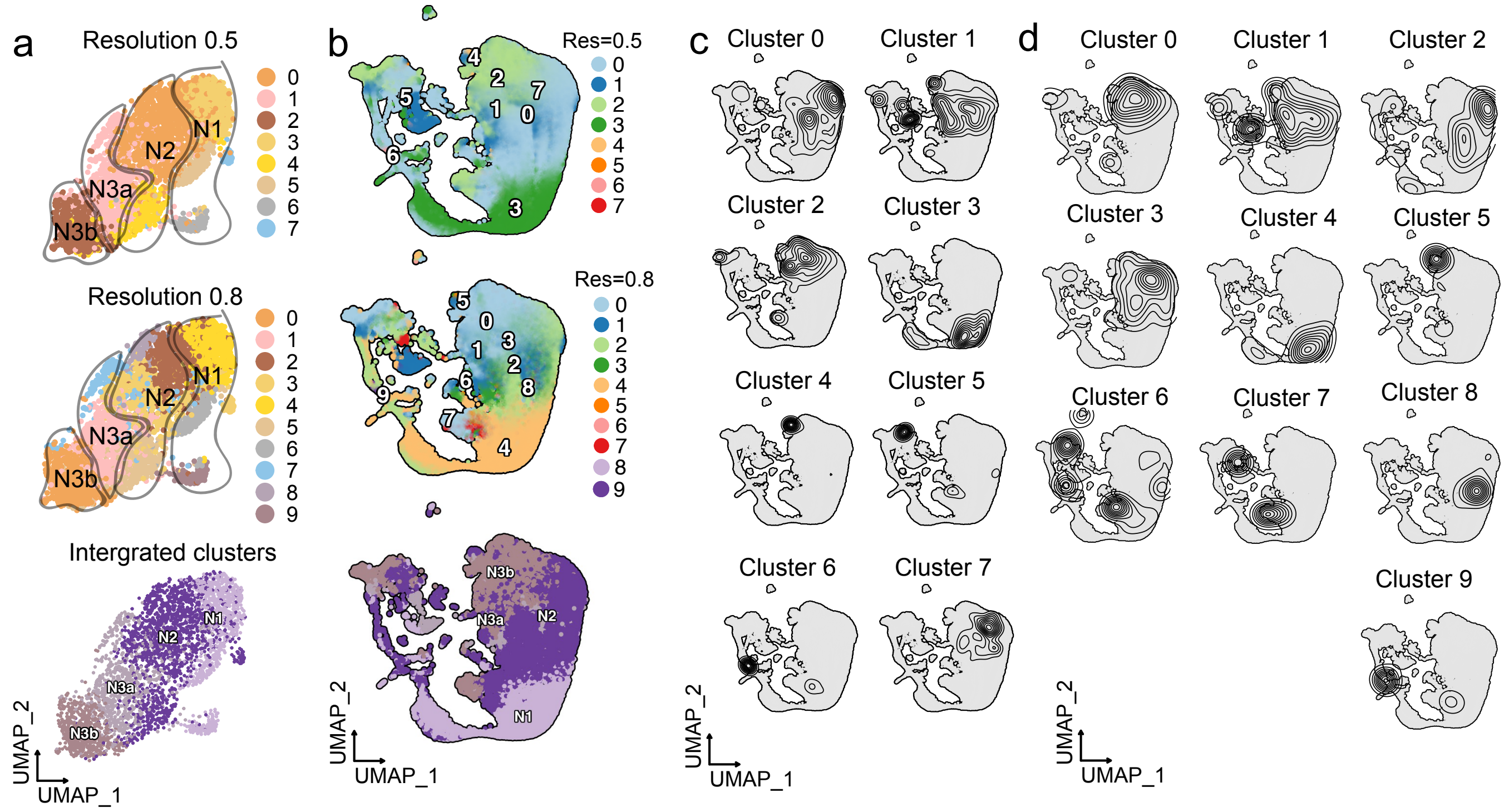

Fig. S4. Projection of fine-grained neutrophil clusters onto NeuMap. (a) UMAP distributions of refined neutrophil clusters at resolution 0.5 (upper) and resolution 0.8 (middle), and the merged neutrophil subsets N1–N3b (bottom). (b) NeuMap projections comparing the clusters resolution 0.5 and 0.8, with the merged neutrophil subsets N1–N3b. Cluster labels mark the centers of the corresponding cell groups. (c–d) Density plots showing the distributions of neutrophil clusters identified at resolutions 0.5 and 0.8, respectively, in NeuMap. Contours denote the areas encompassing 85% of cells.

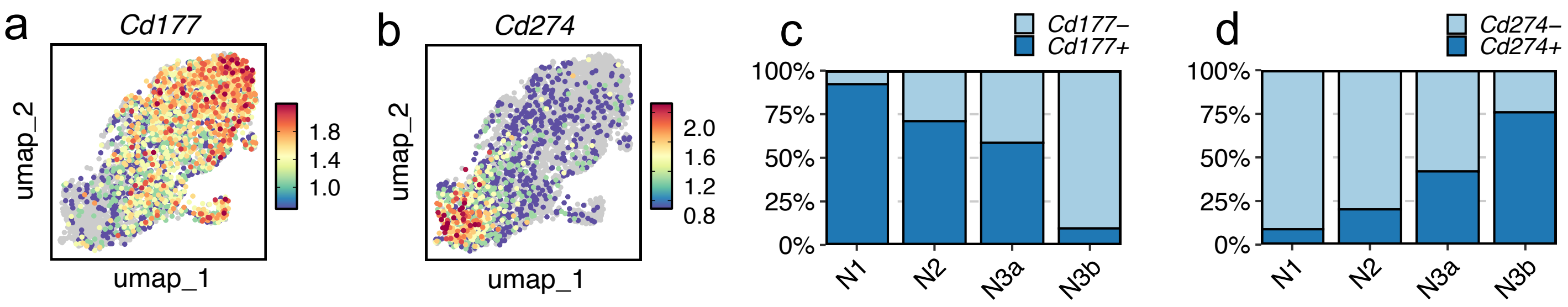

Fig. S5. Distribution of *Cd177*⁺ cells and *Cd274*⁺ cells. (a-b) UMAP plot showing the expression distribution of *Cd177* and *Cd274* in neutrophils. (c-d) Percentages of *Cd177*⁺ cells and *Cd274*⁺ cells in N1, N2, N3a, and N3b.

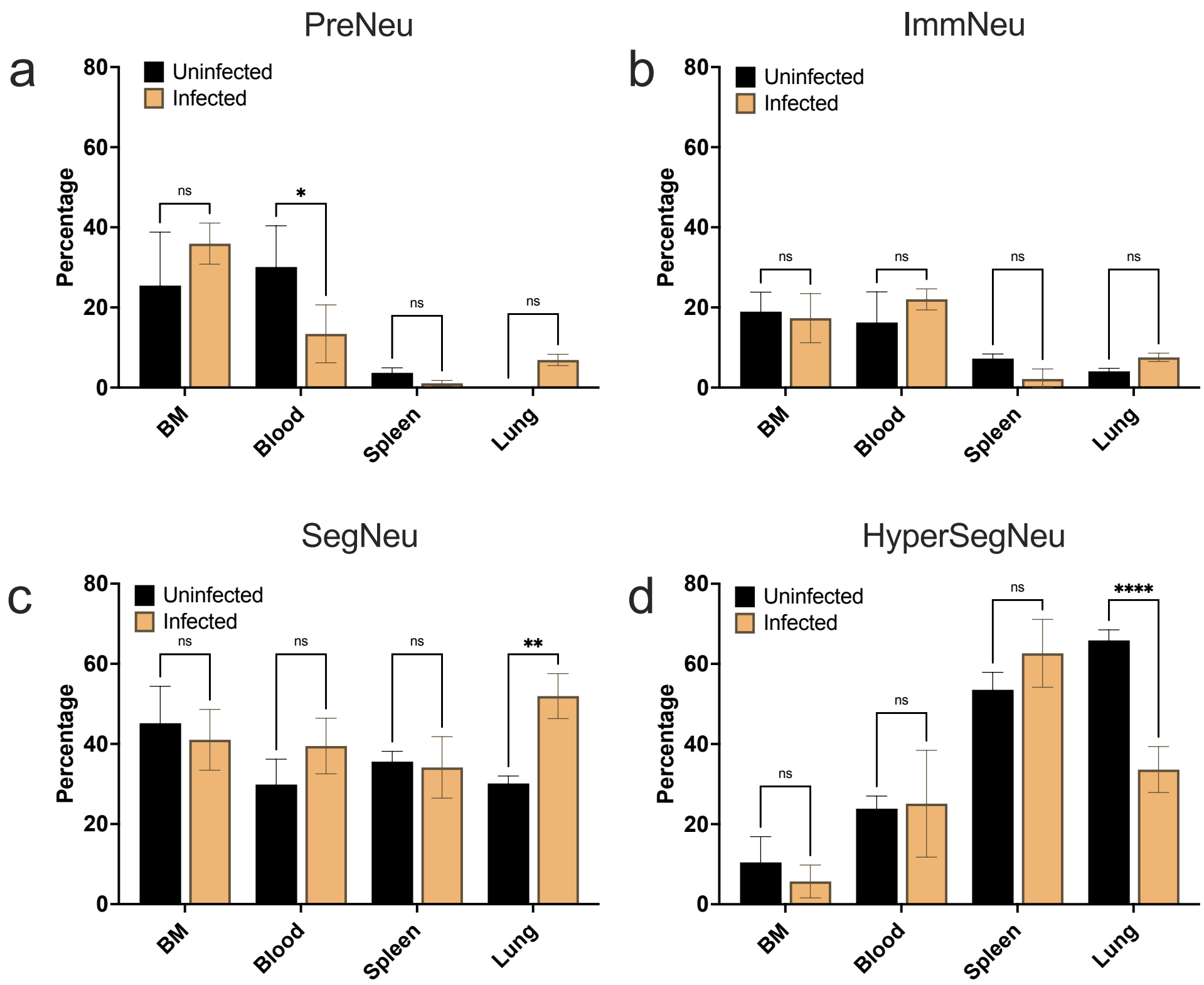

Fig. S6. Comparison of neutrophil morphological proportions in different tissues. (a-d) comparisons of neutrophil morphologies (PreNeu, ImmNeu, SegNeu, and HypersegNeu) across distinct anatomical compartments under infected or uninfected condition, including the bone marrow, peripheral blood, lung, and spleen. Data are presented as mean ± SD (n = 3-4 mice per group). Statistical significance was

determined separately for each morphology using two-way ANOVA with infection status and tissue type as factors, followed by Sidak's multiple comparisons test to compare infected and uninfected groups within each tissue. *$p < 0.05$, **$p < 0.01$, ****$p < 0.0001$.
