# Supplementary material for: Single‐Cell Atlas Reveals Neutrophil Heterogeneity in Hypervirulent Klebsiella pneumoniae Infection‐Induced Sepsis
Source: Immunology. 2026 Aug 27;179(4):624–34. doi: 10.1111/imm.70193 (PMC13725439; doi:10.1111/imm.70193)
Supplement: Supplementary file 2 [file IMM-179-624-s002.docx]

**Supplementary tables**

**Table S1.** Top 20 gene markers of neutrophil subsets.

| Subset | N1 | N2 | N3a | N3b |
| --- | --- | --- | --- | --- |
| Genes | *Ltf* | *D8Ertd738e* | *Asprv1* | *Ccl3* |
|  | *Camp* | *Dgat1* | *Gbp2* | *Gadd45b* |
|  | *Serpinb1a* | *Retnlg* | *Ifi204* | *Tnf* |
|  | *Ngp* | *Mmp8* | *Fas* | *Bcl2a1b* |
|  | *Syne1* | *Adam8* | *Wfdc17* | *Cd274* |
|  | *Chil3* | *Ndel1* | *F630028O10Rik* | *Ccl4* |
|  | *Sec61g* | *Ell2* | *Pla2g7* | *Marcks* |
|  | *Anxa1* | *Stk17b* | *Rab11fip1* | *Nr4a1* |
|  | *Hmgn2* | *Mmp9* | *Fpr1* | *Ly6i* |
|  | *Tkt* | *Thbs1* | *Stat1* | *Bri3* |
|  | *Cybb* | *Rdh12* | *Snx10* | *Klf2* |
|  | *Arhgdib* | *Slc25a3* | *Sell* | *Il1a* |
|  | *Cd177* | *Picalm* | *Oasl2* | *Ninj1* |
|  | *Anxa3* | *Entpd1* | *Upp1* | *Ccrl2* |
|  | *Rasa2* | *Rps27a* | *Sppl2a* | *Nfkbia* |
|  | *Pdcd6* | *Slc16a3* | *Parp14* | *Ptgs2* |
|  | *Sec61b* | *Rpl23* | *Ifitm1* | *Icam1* |
|  | *H2afz* | *Myh9* | *Samhd1* | *Mpeg1* |
|  | *Dstn* | *Serf2* | *Lcp2* | *Sqstm1* |
|  | *Ifitm6* | *Actg1* | *Icam1* | *Cxcl2* |

**Table S2.** Neutrophil functional score genes.

| ROS | *Abcg2*, *Duox1*, *Inava*, *Acox1*, *Cysltr1*, *Lipa*, *Ncf1*, *Duox2*, *Letmd1*, *Blvrb*,*Cldn17*, *Duoxa1*, *Blvra*, *Cyp1a2*, *Cyp1a1*, *Cybb*, *Duoxa2*, *Duox1*, *Ahr*, *Abcc9*, *Mpo*, *Abcc1*, *Cyba*, *Sod2*, *Sod1*, *Dmd*, *Gbf1* |
| --- | --- |
| Activation | *Ccl5*, *Fcer1g*, *Cxcl5*, *Vamp7*, *Gkn2*, *Tyrobp*, *Prg3*, *Cxcr2*, *Bcr*, *Camp*, *Il18rap*, *Myd88*, *Pikfyve*, *F2rl1*, *Prkcd*, *Il15*, *Fcgr4*, *Anxa1*, *Scnn1b*, *Dnase1l3*, *Il18*, *Stx11*, *Anxa3*, *Dnase1*, *Myo1f*, *Traf3ip2*, *Syk*, *Stxbp2*, *Stxbp3*, *Abr*, *Kmt2e*, *Ctsg* |
| Chemotaxis | *Dpep1*, *Ccl3*, *Ccl2*, *Fcer1g*, *Cxcl5*, *Cxcl17*, *S100a8*, *C5ar1*, *Bsg*, *Slc37a4*, *Gm5849*, *Csf3r*, *Cxcl13*, *Ccl28*, *Trem1*, *Trem3*, *Gbf1*, *Cxcl15*, *Cxcr2*, *Cxcl3*, *Cxcl2*, *mCCL27*, *Vav3*, *Spp1*, *Itgb2l*, *S100a9*, *Pikfyve*, *Cxcr1*, *Gpr35*, *Cklf*, *Cxcl9*, *Ppib*, *Ppia*, *Prkca*, *Prex1*, *Itga9*, *Lgals3*, *Itgb2*, *Itgam*, *Itga1*, *mSRP54*, *Pf4*, *Ppbp*, *Cxcl1*, *Jaml*, *Il17b*, *Cxadr*, *Il1b*, *Ifng*, *Pde4b*, *Pde4d*, *Syk*, *Edn3*, *Edn2*, *Cx3cl1*, *Vav1*, *Cxcl10*, *Tgfb2*, *Fcgr3* |
| Glycolysis | *S100a5*, *3110039M20Rik*, *Dlx2*, *Gm25857*, *Mir7056*, *ENSMUSG00000100868*, *Gm26184*, *ENSMUSG00000095116*, *Gm42912*, *Mir130a*, *ENSMUSG00000100788*, *Gm9399*, *Mir877*, *ENSMUSG00000096538*, *ENSMUSG00000094030*, *ENSMUSG00000097652*, *Gm3646*, *Mir133a-2*, *Trbd1*, *Gm47290*, *Mir1b*, *Gm29346*, *mt-Td*, *Traj18*, *Defb29*, *Atp6v1g3*, *Gm12121*, *Mir196a-1*, *Gm12678*, *Cldn16*, *Gm19541*, *Mir6364*, *Mup10*, *Mup21*, *Urad*, *Gm12909*, *Gm13773*, *ENSMUSG00000100318*, *Mup16*, *Mup2*, *P3r3urf*, *Txndc8*, *4930403D09Rik*, *Banf2*, *Gm5941*, *Ctxnd2*, *Mir468*, *Gm9059* |
| Phagocytosis | *Mfge8, Msr1, Aif1, Fcer1g, Fcgr1, Xkr7, Vamp7, Trem2, Timd4, Bin2, Gulp1, Becn1, Adgrb1, Timd2, Havcr1, Sirpa, Ighg3, Xkr4, Arhgap12, Clec7a, Treml4, Rac1, Dppa1, Clcn2, Sh3bp1, Itgb2, Itgam, Marco, Megf10, Gsn, Arhgap25, Xkr8, Elmo1, Clcn3, Ighg1, Ighg2b, Ighg2a, Timd5, Myh9, Xkr6, Abca1, Timd6, Cdc42, Thbs1, Fcgr3* |
| MDSC  (Alshetaiwi *et al.*, 2020) | *Wfdc17*, *Ifitm1*, *Il1b*, *BC100530*, *Srgn*, *Gm5483*, *Stfa2l1*, *Stfa2*, *Prok2*, *Ifitm2*, *Junb*, *Dusp1*, *Socs3*, *Btg1*, *1600014C10Rik*, *Selplg*, *Asprv1*, *Igfbp6*, *Gm5150*, *Pla2g7*, *Csf3r*, *Cxcr2*, *Tpd52*, *Tspo*, *Cyp4f18*, *Grina*, *Fabp5*, *Clec4d*, *Steap4*, *Map1lc3b*, *Ccr1*, *Lrg1*, *Clec4e*, *Ctsd*, *Cd84*, *Gcnt2*, *Arg2*, *BC117090*, *Npl*, *Fgl2*, *Rnf149*, *Sephs2*, *S100a6*, *Lmnb1*, *Amica1*, *Eif4ebp1*, *Msrb1*, *Ubb*, *C5ar1*, *Ypel3*, *Fcgr3*, *Gsr*, *Taldo1*, *Atp6v1g1*, *S100a11*, *Hp*, *Alox5ap*, *Litaf*, *Txn1*, *Upp1*, *AB124611*, *Gpcpd1*, *Snap23*, *Il4ra*, *Hist1h2bc*, *Retnlg*, *Myd88*, *Adipor1*, *Stk17b*, *Zyx*, *Rgs3*, *Il1f9*, *Hdc*, *Atg3*, *Gda*, *Slc40a1*, *Tarm1*, *Cdk2ap2*, *Stfa3*, *Glipr2*, *Tacstd2*, *Picalm*, *Mtus1*, *Fbxl5*, *Slfn1*, *Rnd1*, *Ier2*, *Mxd1*, *Siglece*, *Cdkn2d*, *Cd33*, *Lilr4b*, *Cd14*, *Sell*, *Ppt1*, *Skap2*, *Sfxn5*, *2810474O19Rik*, *Ikbkap*, *Hbb-bs*, *Atp11b*, *Cxcl2*, *Osm*, *Ier3*, *Hba-a1* |
